# Optimization of Low-Density Hydroceramic Systems for Long-Term Stability at High Temperatures

**DOI:** 10.3390/ma18040841

**Published:** 2025-02-14

**Authors:** Chuangchuang Wang, Xueyu Pang, Xiujian Xia, Yongjin Yu, Kaihe Lv, Jinsheng Sun

**Affiliations:** 1 State Key Laboratory of Deep Oil and Gas, China University of Petroleum (East China), Qingdao 266580, China; b21020008@s.upc.edu.cn (C.W.); lkh54321@126.com (K.L.); 2 School of Petroleum Engineering, China University of Petroleum (East China), Qingdao 266580, China; 3 CNPC Engineering Technology R&D Co., Ltd., Beijing 102206, China; xiaxiujiandr@cnpc.com.cn (X.X.); yuyongjindri@cnpc.com.cn (Y.Y.)

**Keywords:** hydroceramic systems, Ca/Si/Al ratio, strength retrogression, macroscopic properties, microscopic properties

## Abstract

In this study, various raw materials, including silica sand, silica fume, calcium hydroxide, α-alumina, and nano-activated alumina, were used to produce hydroceramic systems with varying Ca/Si/Al ratios to optimize their high-temperature resistance. The hydroceramic slurries, with a constant density of 1.65 g/cm^3^, were all designed to have a setting time of more than 4 h at the condition of 240 °C and 50 MPa and then cured at the same condition for 2, 30, and 90 days to evaluate their long-term performances. Subsequently, compressive strength, water permeability, mercury intrusion porosimetry, thermogravimetry, and X-ray diffraction tests were conducted on set samples at various curing times to analyze the hydroceramic systems’ long-term stability and the underlying mechanism. The results indicated that the hydration reaction of α-Al_2_O_3_ was minimal, and its inclusion reduced the incorporation of silica sand in the hydration process. Nano-activated alumina improved the macroscopic properties of the hydroceramic systems and promoted the formation of a significant amount of tobermorite 11 Å. The addition of silica fume can enhance the system’s macroscopic properties and the long-term stability, promoting the reaction of silica sand. The long-term stability of slurries with a Ca/Si ratio of 1 was significantly better than that of slurries with a Ca/Si ratio of 0.5. The best-performing slurry can maintain a compressive strength of more than 19 MPa after being cured at 240 °C for 90 days.

## 1. Introduction

A substantial reserve of deep and ultra-deep oil and natural gas has been identified in China, with a total estimated at 67.1 billion tons of oil equivalent, constituting 34% of the nation’s total oil and gas resources [1,2]. The significant potential of the development of these oil and gas resources has led to an increase in the number of deep (depths exceeding 4500 m) and ultra-deep wells (depths exceeding 6000 m) in recent years. Between 2016 and 2020, the number of these wells rose by 222% and 115%, respectively [3]. Currently, some ultra-deep wells reach depths exceeding 8000 m, with bottom-hole temperatures reaching up to 240 °C. Conventional oil well cement systems can hardly withstand such elevated temperatures. During hydration reaction, the conventional oil well cement forms considerable quantities of calcium–silicate–hydrate (C-S-H). Above 110 °C, this amorphous C-S-H gel transitions into a semi-crystalline or crystalline mineral phase, reducing cement strength and increasing water permeability—a process known as cement strength retrogression [4,5]. Above 150 °C, the severity of this retrogression sharply escalates, compromising wellbore integrity [5]. A common remedy involves incorporating substantial silica into the cement slurry to adjust the calcium–silicon ratio, thus mitigating the strength retrogression. Notwithstanding, studies reveal that conventional density silica-enriched cement systems, when cured for 30 days at 200 °C, exhibit strength retrogression ranging from 27% to 44% [6,7], which can exceed 60% after 90 days of curing [6]. In low-density systems, this retrogression is notably more pronounced [8,9,10,11].

Hydroceramic systems, composed of raw materials, including CaO, Ca(OH)_2_, SiO_2_, Al_2_O_3_, MgO, and Mg(OH)_2_, alongside water, undergo hydration reactions in high-temperature hydrothermal conditions, yielding hydration products with excellent high-temperature stability. Current hydroceramic systems can generally be categorized into CaO-SiO_2_-H_2_O systems [12,13], CaO-Al_2_O_3_-SiO_2_-H_2_O systems [14,15,16], and CaO-MgO-SiO_2_-H_2_O systems [17], among others. Research on the CaO-SiO_2_-H_2_O system mainly focuses on hydration products. Bernstein et al. [18] studied the effect of quartz particle size on the formation of 1.13 nm tobermorite in the hydration products in the CaO-SiO_2_-H_2_O system under hydrothermal conditions of 210 °C using silica sand with particle sizes of 8 μm and 16 μm. The results indicate that 1.13 nm tobermorite is formed through the transformation of semi-crystalline CSH phases in the hydration products, and finer silica accelerates the hydration reaction. Dambrauskas et al. [12] explored the reaction process of the CaO-SiO_2_-H_2_O system with a Ca/Si molar ratio of 1.5 under hydrothermal conditions of 175 °C. The results indicate that within 48 h of the reaction, Ca(OH)_2_ reacts completely, and semi-crystalline CSH phases transform into crystalline α-C_2_SH. Baltakys et al. [19] investigated the influence of reaction time on the crystallinity and stability of gyrolite in the hydration products of the CaO-SiO_2_-H_2_O system with a Ca/Si molar ratio of 0.66 under hydrothermal conditions of 200 °C. The results demonstrate that after 120 h to 168 h of reaction time, gyrolite is the most stable. Research on CaO-Al_2_O_3_-SiO_2_-H_2_O systems is quite comprehensive, covering aspects such as phase equilibrium, the impact of temperature and pressure on hydration products, the influence of the Ca/Si ratio on hydration products, the effect of different raw materials on hydration products, and the impact of the particle size of raw materials on hydration products. Under hydrothermal conditions of 200~350 °C, the hydration products of the CaO-Al_2_O_3_-SiO_2_-H_2_O system include a variety of minerals, such as gyrolite, gillebrandite, jaffeite, etc., with the type of minerals formed being closely related to the Ca/Si ratio as well as temperature and pressure conditions [20]. When α-alumina is used as the aluminum source, the high-temperature stability of tobermorite 11Å can be greatly enhanced. Adding γ-Al_2_O_3_ to the system stimulates the formation of C-S-H [19,21]. Using finer silica in the system promotes the formation of hydrogarnet [22]. Research on the CaO-MgO-SiO_2_-H_2_O system has been limited. It has been demonstrated that optimal performance of the system occurs when the mass ratio of CaO to MgO to SiO_2_ is 1:1:2. Furthermore, incorporating K_2_O and Na_2_O enhances the cementation properties of the system [17]. From the above studies, some researchers have proposed that hydroceramic systems can be used as substitutes for cement systems in cementing operations in deep wells or geothermal wells [23,24].

Research on hydroceramic systems has primarily produced theoretical results despite extensive study. These systems are noted for their excellent high-temperature stability, yet real-world applications remain under-researched. This study aims to develop hydroceramic systems consisting of varying raw material compositions and Ca/Si/Al ratios, targeting their potential use in cementing operations in deep wells. Since raw materials are all powdery materials with high specific surface area, the slurry density was set at 1.65 g/cm^3^, aligning it within the range typical for low-density cementing fluids. The reaction time of the slurries was optimized, and then, they were cured at 240 °C/50 MPa for 2, 30, and 90 days. Subsequent evaluations focused on the macroscopic and microscopic properties, analyzing factors that influence long-term stability and delineating the high-temperature resistance mechanisms of the system. These results also have certain guiding significance for the current design of high-temperature-resistant cement systems.

## 2. Materials and Experimental Procedure

### 2.1. Raw Materials

In this study, five raw materials were used for the design of the systems, including calcium hydroxide (CH) produced by China National Pharmaceutical Group Corporation (Shanghai, China), silica sand (SL) produced by Henan Tongbai Factory (Nanyang, China), silica fume (SF) produced by Xinjiang Kuche Drilling Mud Material Factory (Kuche, China), α-alumina (α-Al) produced by Guangdong Senxin Co., Ltd. (Guangzhou, China), and nano-activated alumina (Nano-Al) produced by Hubei Huifu Nanomaterial Co., Ltd. (Yichang, China). The composition, particle size, and density of these materials are shown in Table 1. To optimize the engineering properties of the systems, several chemical additives were chosen, including a suspension aid (SUS), a retarder (RET), and a fluid loss agent (FLU) from OMAX Oilfield Technology Co., Ltd. (Chengdu, China), a dispersant (DIS) from Weihui Chemical Co., Ltd. (Weihui, China), and a defoamer (DEF) from Tianjin PetroChina Boxing Technology Co., Ltd. (Tianjin, China).

### 2.2. Slurry Composition Design

To optimize the long-term stability of hydroceramic systems, two series of slurries, namely the ‘L’ series and the ‘H’ series, were designed using calcium hydroxide (CH), silica sand (SL), silica fume (SF), α-alumina (α-Al), and nano-activated alumina (Nano-Al). The Ca/Si ratio of the slurry ‘L’ series was 0.5, while the ratio of the slurry ‘H’ series was 1. The Ca/Si ratios of the two series of slurries were different, but both contained the following six combinations of raw materials: CH+SL (CS), CH+SL+α-Al (CSA), CH+SL+α-Al+Nano-Al (CSAA), CH+SL+SF (CSS), CH+SL+SF+α-Al (CSSA), and CH+SL+SF+α-Al+Nano-Al (CSSAA). The curing autoclave used in the subsequent curing experiments required 2.5 h to reach the curing conditions of 240 °C/50 MPa. To ensure all slurries underwent hydration reactions at the target curing conditions, the dosage of retarder was adjusted so that the hydration reaction times of all slurries exceeded 2.5 h. Additionally, the suspension stability, pumpability, and fluid loss control of the slurries were adjusted according to API-10B-2 [25] using the suspension aid, dispersant, and fluid loss agent. Finally, an optimal amount of defoamer was also incorporated to minimize bubble content. The detailed compositions of the twelve slurries are shown in Table 2.

### 2.3. Hydration Reaction Time Test

According to the requirements of curing experiments on the hydration reaction time of slurries, it was necessary to optimize the dosage of retarders to ensure that the hydration reaction time of all slurries exceeded 2.5 h. Additionally, since the hydration reaction of slurries releases heat [26], the hydration reaction time can be evaluated by testing the time of heat release of slurries under the target testing conditions. A C80 microcalorimeter produced by Setaram, Lyon, France, was used for measuring hydration heat, with temperature and pressure increasing at rates of 2 °C/min and 1.67 MPa/min, respectively, and the final test conditions were 240 °C/50 MPa. During the test, the heat flow within the device was measured by the microcalorimeter in real time. When an increase in heat flow was observed, it indicated that the slurry had begun the hydration reaction, releasing heat, thereby determining the hydration reaction time of the slurry. By changing the dosage of the retarder, the hydration reaction times of all slurries were optimized, thereby determining the detailed compositions of all slurries for subsequent experiments.

### 2.4. Sample Preparation and Physico-Mechanical Property Test

All designed slurries were mixed using a blender manufactured by Tianjin Nithons Technology Co., Ltd. (Tianjin, China), following the standard procedures detailed in API-10B-2 [25]. The mixtures were initially blended at a speed of 4000 rpm for 15 s, followed by a higher speed of 12,000 rpm for 35 s. After mixing, the prepared slurries were poured into special molds used for high-temperature curing experiments (mold inner diameter of 2.5 mm and height of 70 mm). Subsequently, they were placed in a curing autoclave with set curing conditions of 240 °C and 50 MPa (according to API-10B-2 [25], the pressure for pressurized curing should exceed 21 MPa, and the maximum pressure capacity of the curing autoclave is 60 MPa), and the curing times were 2 days, 30 days, and 90 days. After the curing experiments, the samples were removed from the molds, and the ends of each sample were appropriately trimmed using a cutting machine, then polished to the standard core size (diameter of 25 mm and height of 50 mm) for physico-mechanical property tests, including compressive strength tests and water permeability tests. To ensure the reliability of the results, three replicate samples from each slurry were subjected to compressive strength tests, and two replicate samples were subjected to water permeability tests.

### 2.5. Mercury Intrusion Porosimetry Test

The research indicates that when the compressive strength of samples significantly decreases, an increase in pore size can also be observed [5]. Therefore, a Quantachrome mercury intrusion pore size analyzer (Model PM 33, Quantachrome, Boynton Beach, FL, USA) was used to assess the porosity and pore size distribution of samples at various curing times. Samples needed to be thoroughly dried in a vacuum dryer before the experiment. One sample from each slurry was selected for the mercury intrusion porosimetry (MIP) test according to standard experimental protocols. After the experiment, the porosity and pore size distribution of slurries at various times were obtained.

### 2.6. Thermogravimetry Test

When hydroceramic systems experience significant strength retrogression, the composition and quantities of hydration products in the samples change. Thermogravimetry analysis (TGA) measures the bound-water content and approximates mineral types by analyzing the temperature ranges of the decomposition peaks. The TGA utilized was a Setaram thermal analysis instrument (Model Setline STA, Setaram, Lyon, France). Samples processed for the experiment were finely ground in an agate mortar after drying in a vacuum dryer at room temperature. Subsequently, samples weighing approximately 70–80 mg were measured using an external balance with a resolution of 0.1 mg. Then, the samples were placed into the equipment and were heated to 105 °C at a rate of 5 °C/min in a nitrogen atmosphere, maintained at this temperature for one hour, and then heated to 1000 °C at the same heating rate. During the heating process, changes in the mass of the samples were recorded via a balance within the equipment, which has a resolution of 0.05 micrograms.

### 2.7. X-Ray Diffraction Test

To analyze the changes in the types and contents of minerals in the sample more accurately, it is necessary to perform X-ray diffraction (XRD) testing on samples at various curing times. The experiment used a Panalytical diffractometer (Model Aeris, Panalytical, Shanghai, China) with a scanning angle of 7º to 70º (2θ angle). The sample preparation before the experiment was the same as for the TGA test. After the experiment, a quantitative analysis of the test results was required. Quantitative X-ray diffraction analysis was performed on the test results using the external standard method and Rietveld refinement. Using monocrystalline silicon as an external standard, combined with the TGA test results of the total water content of the samples, Rietveld refinement integrated in the Highscore Plus 5.0 software allowed for the quantification of varying crystalline minerals, amorphous phases, and free water content within the samples. It facilitated an understanding of the evolution of hydration products in the samples as the curing time increased, which is crucial for exploring the strength retrogression mechanisms of hydroceramic systems. Current research indicates that strength retrogression in the samples involves changes in internal phase compositions, primarily including the transformation from amorphous C-(A)-S-H gel to the crystalline form of xonotlite [27]; an increase in the amounts of crystalline tobermorite and xonotlite [5]; the conversion of tobermorite to xonotlite [28]; and the ongoing consumption of silica sand, resulting in an increase in the total amounts of hydration products [5].

## 3. Test Results and Analysis

### 3.1. Hydration Reaction Time Test Results

**Figure 1:**
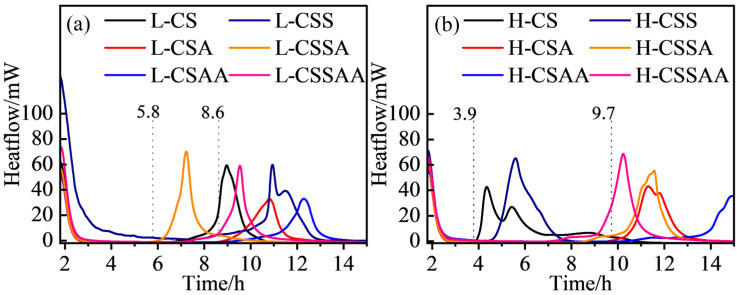
Hydration reaction time of all slurries: (**a**) hydration reaction time of slurry ‘L’ series; (**b**) hydration reaction time of slurry ‘H’ series.

The effects of retarder dosage on the induction time of each slurry were tested under conditions of 240 °C/50 MPa, and the appropriate dosage of retarder for each slurry was determined, as shown in Figure 1. As seen from the figure, when the Ca/Si ratio of the slurry was 0.5, the addition of silica fume greatly accelerated the hydration reaction, thus necessitating a higher dosage of retarder; however, when the Ca/Si ratio of the slurry was 1, this phenomenon was not pronounced. The hydration reaction time of all slurries exceeded 2.5 h, meeting the requirements for subsequent curing experiments. Subsequently, the specific compositions of all slurries were determined and are presented in Table 2.

### 3.2. Physico-Mechanical Property Test Results

**Figure 2:**
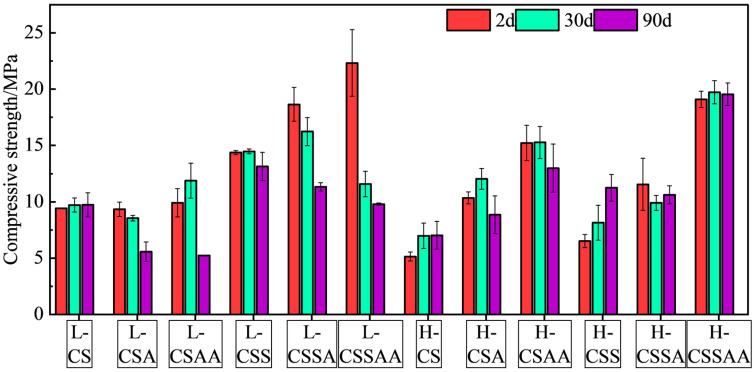
Compressive strength test results of all slurries at various curing times.

**Figure 3:**
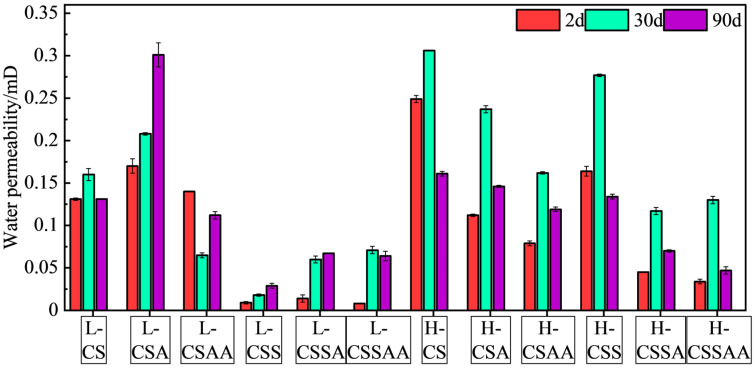
Water permeability test results of all slurries at various curing times.

The compressive strength and water permeability of the twelve designed slurries were tested after curing for 2 days, 30 days, and 90 days under conditions of 240 °C and 50 MPa. The results are shown in Figure 2 and Figure 3, respectively, with error bars representing the standard deviation of the test data, indicating that the experimental errors are relatively insignificant.

As shown in the figures, in slurry ‘L’ series, the compressive strengths of slurries L-CS and L-CSS remained stable during the 90-day curing period, and the changes in their corresponding water permeabilities were also minimal. It indicated that these two systems exhibited good long-term stability. Slurry L-CSS, which included silica fume added to the base of slurry L-CS, suggested that silica fume could enhance the system’s compressive strength and significantly decreased its water permeability without affecting its high-temperature resistance. Slurries L-CSA and L-CSAA, which sequentially added α-alumina (α-Al) and nano-activated alumina (Nano-Al) to the base of slurry L-CS, showed negligible effects on the system’s compressive strength and permeability. However, slurries L-CSA and L-CSAA could only maintain stable compressive strength for 30 days; after curing for 90 days, they exhibited significant strength retrogression, with a decrease of 40% to 47% compared to the set samples cured for 2 days. Slurries L-CSSA and L-CSSAA, based on slurry L-CSS with additions of α-Al and nano-Al, respectively, showed compressive strengths significantly higher than those of slurries L-CS, L-CSA, and L-CSAA after two days of curing. It indicated that the presence of the silica fume in the system likely enhanced the participation of α-Al and nano-Al in the hydration reaction, thereby increasing the system’s compressive strength and decreasing its permeability. Slurry L-CSSA could essentially maintain its compressive strength stably for up to 30 days, but after curing for 90 days, the strengths of set samples decreased by 39% compared to those cured for 2 days. Slurry L-CSSAA had the highest compressive strength of all slurries at 2 days, reaching 22.3 MPa. However, its compressive strength decreased by about 50% after 30 days of curing and 56% after 90 days of curing, marking the largest strength reduction among all the slurries.

In slurry ‘H’ series, only slurries H-CSA and H-CSAA exhibited a 14% strength retrogression after a 90-day curing period compared to the set samples cured for 2 days, and this strength retrogression was lower than that of slurries L-CSA and L-CSAA. Aside from that, the other slurries demonstrated excellent long-term stability, with slurry H-CSS even showing an increase in compressive strength. Similarly, slurries H-CSA and H-CSAA were sequentially added with α-Al and Nano-Al on the basis of slurry H-CS, which resulted in a decrease in long-term stability, but unlike slurries L-CSA and L-CSAA, their addition increased the compressive strength and lowered the water permeability of the slurry. Slurry H-CSS, compared to slurry H-CS added with silica fume, showed a 36% increase in compressive strength after 90 days of curing, representing the best long-term stability among all slurries. Slurry H-CSSAA had the highest compressive strength in slurry ‘H’ series, reaching 19.1 MPa, and showed no strength retrogression after 90 days of curing.

A comprehensive analysis of all slurries revealed that slurries with a Ca/Si ratio of 1 exhibited significantly superior long-term stability than those with a Ca/Si ratio of 0.5. The addition of the silica fume substantially enhanced the macroscopic properties of the slurries. However, adding α-alumina and nano-activated alumina to slurries without silica fume resulted in a decrease in their long-term stability. In slurries containing silica fume, the addition of these components not only further improved the compressive strength but also did not decrease the long-term stability. Furthermore, the density of all the hydroceramic systems in the research was 1.65g/cm^3^, classified as low-density cementing fluids. After curing at 240 °C for 90 days, the most substantial strength retrogression was observed in slurry L-CSSAA with a retrogression magnitude of only 56%, which was still lower than silica-enriched cement slurries under similar conditions, demonstrating the excellent long-term stability of hydroceramic systems [5,7,29].

### 3.3. MIP Test Results

**Figure 4:**
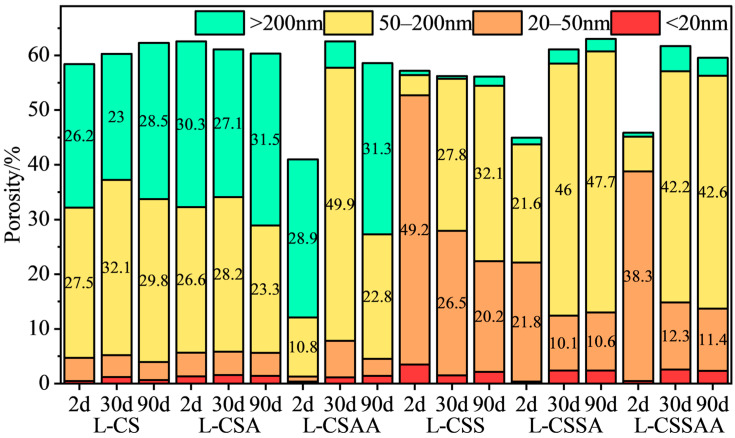
MIP test results of slurry ‘L’ series at various curing times.

**Figure 5:**
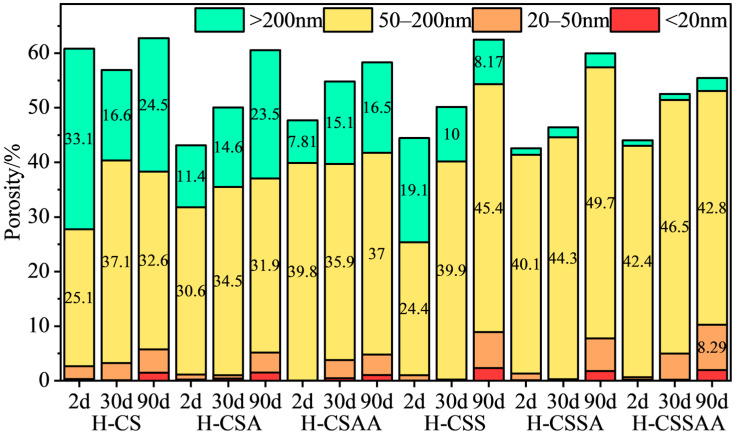
MIP test results of slurry ‘H’ series at various curing times.

The MIP test results of twelve slurries at various curing times are shown in Figure 4 and Figure 5.

Current research has indicated that pores within the sample can be categorized based on their size into more-harmful pores (>200 nm), harmful pores (50–200 nm), less-harmful pores (20–50 nm), and harmless pores (<20 nm) [30]. An increase in the amounts of harmful pores in the sample can lead to a decrease in its macroscopic properties. It can be seen that the total porosity of all slurries at various curing times ranged from 40% to 65%. Slurries L-CS and L-CSA contained larger amounts of more-harmful pores and harmful pores after 2 days of curing. These two slurries had lower compressive strengths, higher water permeability, and larger pore sizes. Prolonged high temperatures had little effect on the amount of harmful pores of these two slurries, making it difficult to determine whether strength retrogression had occurred in these two slurries. As the curing time increased, the amounts of more-harmful pores in slurry L-CSAA first decreased and then increased, which corresponded well with the changes in its compressive strength. With the curing time extended, the amounts of less-harmful pores in slurries L-CSS, L-CSSA, and L-CSSAA decreased, while the amounts of harmful pores in these slurries increased, leading to strength retrogression in all three slurries. Particularly, the significant increase in the amounts of harmful pores in slurry L-CSSAA corresponded to its substantial strength retrogression. With the curing time extended, the amounts of more-harmful pores in slurries H-CSA and H-CSAA increased, leading to strength retrogression in these two slurries. However, the amounts of more-harmful pores in the other four slurries gradually decreased, while the amounts of other types of pores remained stable; thus, they demonstrated long-term stable performance. The MIP test results confirmed that slurries with a Ca/Si ratio of 1 had notably better long-term stability than those with a Ca/Si ratio of 0.5, also demonstrating a close relationship between strength retrogression and the coarsening of pore sizes of the slurries.

### 3.4. TGA Test Results

**Figure 6:**
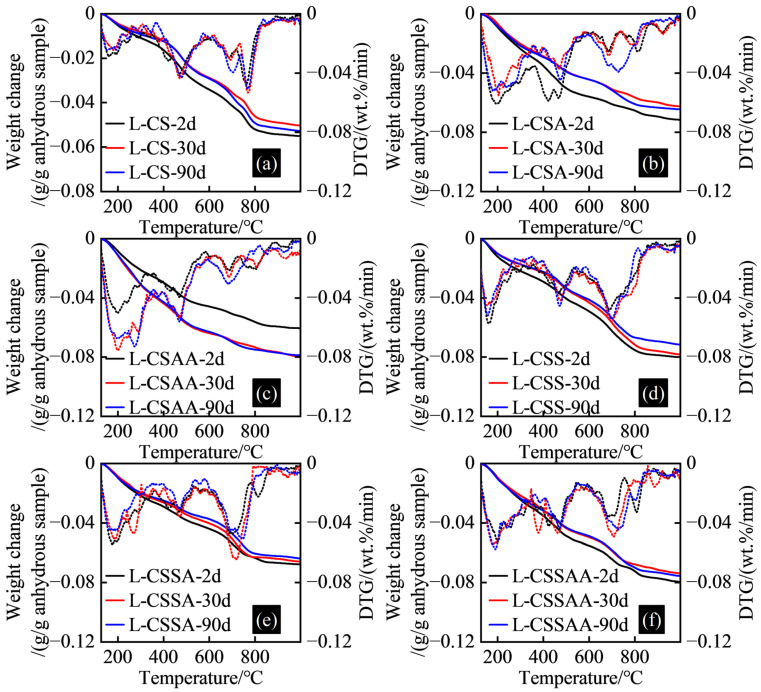
TGA test results of slurry ‘L’ series at various curing times: (**a**) TGA test results of slurry L-CS; (**b**) TGA test results of slurry L-CSA; (**c**) TGA test results of slurry L-CSAA; (**d**) TGA test results of slurry L-CSS; (**e**) TGA test results of slurry L-CSSA; (**f**) TGA test results of slurry L-CSSAA.

**Figure 7:**
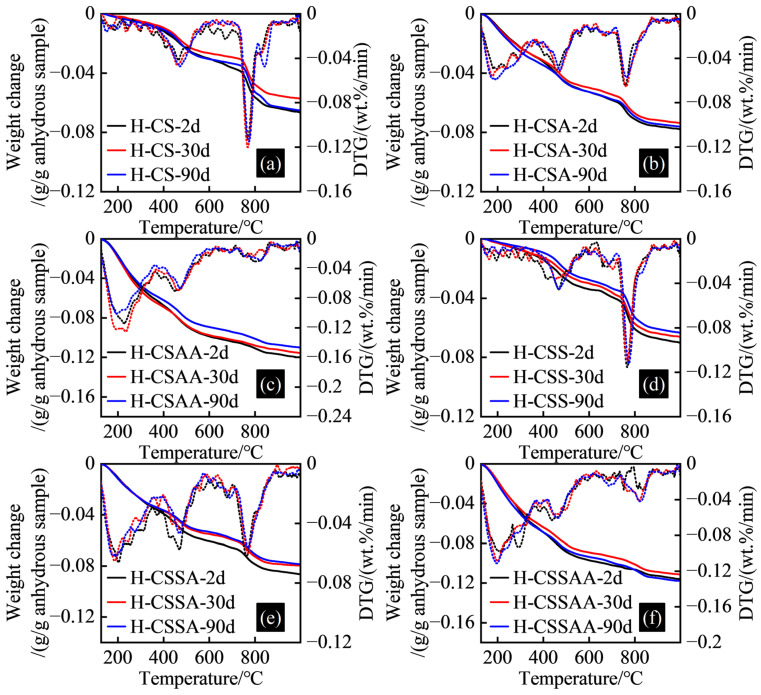
TGA test results of slurry ‘H’ series at various curing times: (**a**) TGA test results of slurry H-CS; (**b**) TGA test results of slurry H-CSA; (**c**) TGA test results of slurry H-CSAA; (**d**) TGA test results of slurry H-CSS; (**e**) TGA test results of slurry H-CSSA; (**f**) TGA test results of slurry H-CSSAA.

The TGA test results of twelve slurries at various curing times are shown in Figure 6 and Figure 7.

The hydration products of hydroceramic systems contain a large amount of C-(A)-S-H, which dehydrates within the temperature range of 105 °C to 600 °C [31]. Under long-term high-temperature curing conditions, the C-(A)-S-H gradually transforms into crystalline or semi-crystalline minerals, as some of the bound water within converts to free water. This transformation is one of the reasons contributing to the strength retrogression of hydroceramic systems [5,27].

The bound-water content in slurry L-CS ranged from 0.05 to 0.055 g/g of the anhydrous sample. The bound-water content in slurry L-CSA, which added α-alumina, increased to 0.063 to 0.072 g/g of anhydrous sample. The bound-water content in slurry L-CSAA, which added α-alumina and nano-activated alumina, was between 0.06 and 0.08 g/g of the anhydrous sample, indicating a gradual increase in the C-(A)-S-H content across these three slurries. The bound-water content in slurry L-CSS ranged from 0.071 to 0.08 g/g of the anhydrous sample. The bound-water content in slurry L-CSSA, which added α-alumina, ranged from 0.064 to 0.068 g/g of the anhydrous sample. The bound-water content in slurry L-CSSAA, which added α-alumina and nano-activated alumina, ranged between 0.074 and 0.08 g/g of the anhydrous sample, suggesting a similar C-(A)-S-H content in these three slurries. As the curing time increased, the bound-water content in the same slurry in slurry ‘L’ series showed little variation.

The bound-water content in slurry H-CS was between 0.057 and 0.067 g/g of the anhydrous sample. The bound-water content in slurry H-CSA, which added α-alumina, increased to 0.074 to 0.078 g/g of the anhydrous sample. The bound-water content in slurry H-CSAA, which added α-alumina and nano-activated alumina, rose to 0.11 to 0.12 g/g of the anhydrous sample, indicating a significant presence of C-(A)-S-H. The bound-water content in slurry H-CSS ranged from 0.063 to 0.07 g/g of the anhydrous sample. The bound-water content in slurry H-CSSA, which added α-alumina, ranged from 0.079 to 0.086 g/g of the anhydrous sample. The bound-water content in slurry H-CSSAA, which added α-alumina and nano-activated alumina, rose to 0.111 to 0.118 g/g of the anhydrous sample, indicating a gradual increase in the C-(A)-S-H content across the three slurries, particularly notable in the hydration products of slurry H-CSSAA. Similarly, the bound-water content in the same slurry in slurry ‘H’ series was minimal. Therefore, based on the current analysis of bound-water content changes within the samples, it was difficult to determine if strength retrogression has occurred, necessitating further testing and analysis.

The DTG analysis results indicated that the primary decomposition peaks occurred within three specific temperature intervals: 105–300 °C, 300–600 °C, and 650–850 °C. Decomposition peaks of tobermorite 11Å (Ca_4_Si_6_O_15_(OH)_2_·5H_2_O, or C_4_S_6_H_6_) in the hydration products ranged from 50 °C to 300 °C and 724°C [32,33], while those of xonotlite (Ca_6_Si_6_O_17_(OH)_2_, or C_6_S_6_H) ranged from 770 °C to 800 °C [32]. Notably, slurries L-CS, L-CSS, and L-CSSA displayed marked decomposition peaks of xonotlite, yet these were more distinct in slurries H-CS, H-CSA, H-CSS, and H-CSSA, suggesting that a higher Ca/Si ratio of 1 led to substantial formation of xonotlite. It was one of the contributing factors to the superior long-term stability observed in slurries in the slurry ‘H’ series. TGA test results of the bound-water content and mineral composition of the samples revealed consistent bound-water levels across all slurries over time. The hydration products in slurries in the slurry ‘H’ series notably contained large amounts of xonotlite, which may enhance their long-term stability. To further investigate the reasons for potential strength regression in the different slurries, a detailed phase analysis of post-reaction samples was required to understand the internal mechanisms based on the alterations in various hydration products.

### 3.5. XRD Test Results

**Figure 8:**
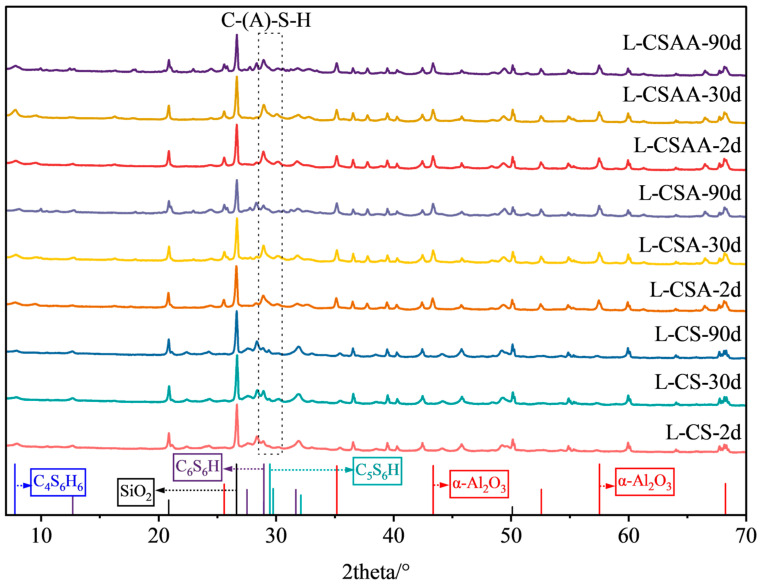
XRD test results of slurries L-CS, L-CSA, and L-CSAA at various curing times.

**Figure 9:**
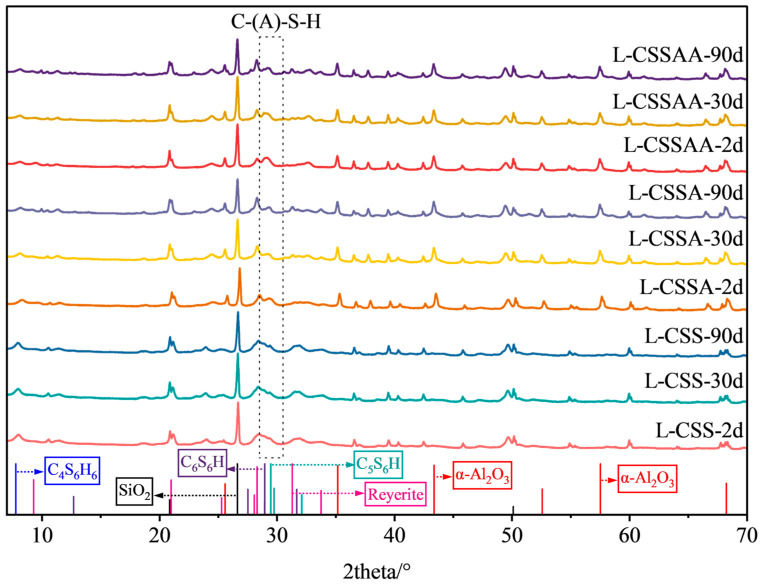
XRD test results of slurries L-CSS, L-CSSA, and L-CSSAA at various curing times.

**Figure 10:**
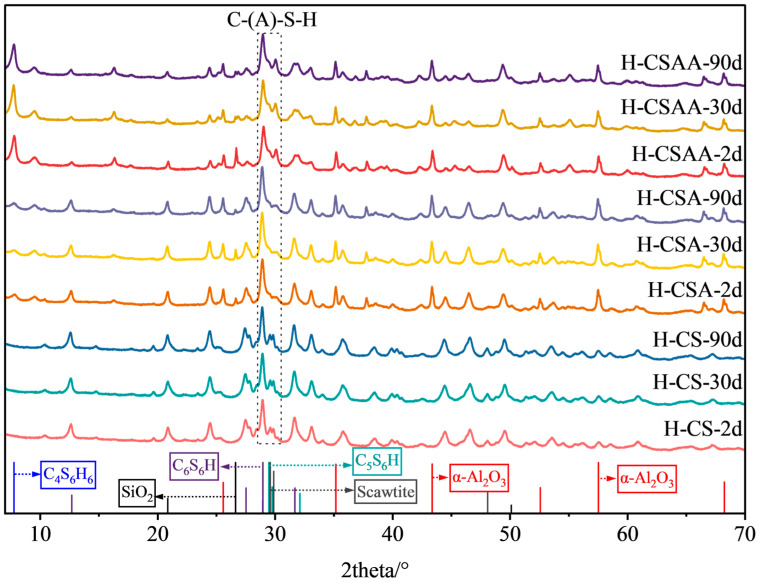
XRD test results of slurries H-CS, H-CSA, and H-CSAA at various curing times.

**Figure 11:**
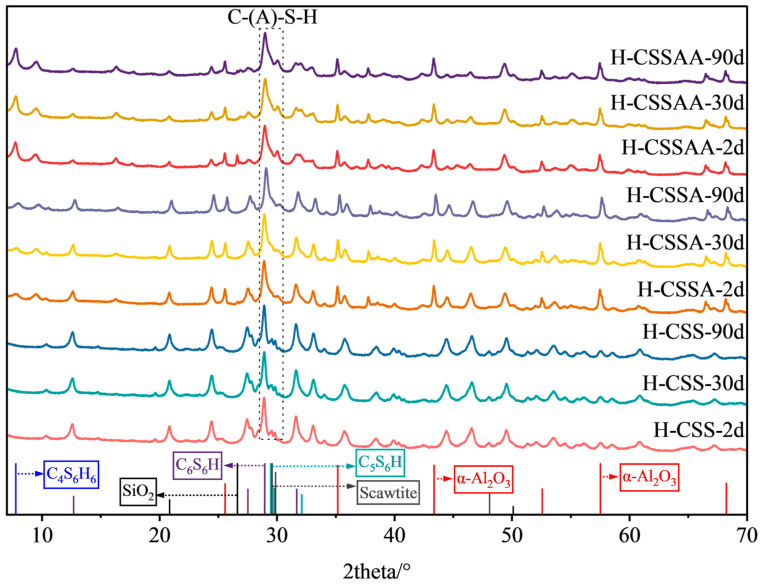
XRD test results of slurries H-CSS, H-CSSA, and H-CSSAA at various curing times.

The XRD qualitative analysis results of twelve slurries at various curing times are shown in Figure 8, Figure 9, Figure 10 and Figure 11. The XRD quantitative analysis results are shown in Table 3, Table 4, Table 5 and Table 6.

The qualitative and quantitative XRD analysis results of slurries L-CS, L-CSA, and L-CSAA are shown in Figure 8 and Table 3. It can be seen that when the Ca/Si ratio was 0.5, the main hydration products of the slurries included C-(A)-S-H gel, tobermorite 9Å (Ca_5_Si_6_O_16_(OH)_2_, or C_5_S_6_H), tobermorite 11Å (Ca_4_Si_6_O_15_(OH)_2_·5H_2_O, or C_4_S_6_H_6_), and xonotlite (Ca_6_Si_6_O_17_(OH)_2_, or C_6_S_6_H) [34,35]. Additionally, there was a large amount of unreacted silica sand and α-alumina remaining. In slurry L-CS, 45.4% of the silica sand participated in the hydration reaction after two days of curing. Subsequently, the amount of silica sand and the total amounts of hydration products remained stable with no new minerals formed, indicating that the hydration reaction was completed in the early stages of hydration. As the curing time increased, the content of tobermorite decreased, while the content of xonotlite increased, indicating a transformation between the two. However, the compressive strength of slurry L-CS remained stable after two days of curing because the hydration products contained a large amount of xonotlite in the hydration products of the slurry. Although its content subsequently increased, it was not enough to cause a decrease in compressive strength. Furthermore, a stable silica sand content and an increase in the C-S-H gel amount contributed to the consistent compressive strength. In slurries L-CSA and L-CSAA, 40.2% and 43.8% of the silica participated in the hydration reaction after two days of curing. Subsequently, the amount of silica sand continuously decreased, and the total amounts of hydration products gradually increased, indicating that the hydration reaction was ongoing. As the curing time increased, the content of α-alumina in the samples remained almost unchanged, indicating that α-alumina hardly participated in the reaction; the content of C-(A)-S-H increased, but the mechanically favorable tobermorite and the high-temperature stable xonotlite were present in minimal amounts, along with the formation of a small amount of new minerals, accounting for the poor high-temperature resistance of these two slurries.

The qualitative and quantitative XRD analysis results of slurries L-CSS, L-CSSA, and L-CSSAA are shown in Figure 9 and Table 4. Slurries L-CSS, L-CSSA, and L-CSSAA were designed by adding silica fume to slurries L-CS, L-CSA, and L-CSAA. In addition to the types of hydration products previously mentioned, a mineral named reyerite ((Na, K)_2_Ca_14_Si_22_Al_2_O_58_(OH)_8_·6H_2_O) [36] was identified in the samples. In slurry L-CSS, the content of silica sand and the total amounts of hydration products remained stable after two days of curing, with no new minerals formed, indicating that the hydration reaction was already completed in the early stages. As the curing time increased, the content of C-S-H slightly decreased, but the content of tobermorite increased, indicating that a small amount of C-S-H was transformed into mechanically favorable tobermorite; thus, slurry L-CSS was still able to maintain long-term compressive strength stability. In slurries L-CSSA and L-CSSAA, the amount of silica sand continued to decrease as the curing time increased. The total amounts of hydration products gradually increased, and crystalline reyerite was formed after the hydration reaction. Notably, after 90 days of curing, the high content of reyerite was the leading cause of the significant strength retrogression in slurries L-CSSA and L-CSSAA.

The qualitative and quantitative XRD analysis results of slurries H-CS, H-CSA, and H-CSAA are shown in Figure 10 and Table 5. It can be seen that when the Ca/Si ratio was 0.5, the main hydration products of the slurries included C-(A)-S-H gel, tobermorite 9 Å, tobermorite 11 Å, xonotlite, and scawtite (Ca_7_(Si_6_O_18_)(CO_3_)·2H_2_O) [37]. In addition to this, a significant quantity of unreacted α-alumina was observed. It indicated that when the Ca/Si ratio was 1, the calcium hydroxide and silica sand fully reacted, illustrating that α-alumina’s role in the hydration process was unaffected by the system’s Ca/Si ratio due to its minimal reactivity, which led to substantial residues after the reaction. In the early stages of the reaction, slurries H-CS and H-CSA generated large amounts of xonotlite. However, the total amounts of hydration products and the content of each component in slurry H-CS changed very little during the 90-day curing period, which was the primary factor contributing to its stable strength. As the curing time increased, a slight decrease in strength was observed in slurry H-CSA, attributable to the formation of new minerals and a slight increase in xonotlite accompanied by a decrease in C-(A)-S-H. In slurry H-CSAA, the primary mineral internally was the mechanically excellent tobermorite. As the curing increased, some of the tobermorite transformed into xonotlite, which also resulted in a slight decrease in strength.

The qualitative and quantitative XRD analysis results of slurries H-CSS, H-CSSA, and H-CSSAA are shown in Figure 11 and Table 6. Slurries H-CSS, H-CSSA, and H-CSSAA were designed by adding silica fume to slurries H-CS, H-CSA, and H-CSAA, and their types of hydration products were exactly the same. Slurries H-CSS and H-CSSA generated a large amount of xonotlite in the early stages of the reaction. As the curing time increased, the content of C-(A)-S-H gradually increased, with no significant new minerals forming, thus these two slurries did not experience strength retrogression. Slurry H-CSSAA contained a large amount of C-(A)-S-H gel, which corresponded well with the TGA test results (see Figure 7). The main hydration product of the slurries was tobermorite after 2 days of curing. As the curing time increased, its content did not decrease, resulting in its stable strength under long-term high-temperature conditions.

By analyzing the changes in the content of each hydration product in slurries at various curing times, it can be concluded that the main characteristics of slurries in hydroceramic systems that maintain stable strength were as follows: the silica sand content remained stable rather than being continuously consumed; no significant new minerals were formed; and a large amount of xonotlite or tobermorite was generated in the early stages of the hydration reaction, and as the curing time increased, their content remained stable.

From the test results, it can be concluded that hydroceramic systems had excellent long-term stability compared to conventional cement systems. The slurries with a Ca/Si ratio of 1 exhibited better high-temperature resistance than those with a ratio of 0.5. The addition of silica fume significantly enhanced the macroscopic properties of the slurries, as well as the participation of silica sand in chemical reactions in the system. The reactivity of α-Al_2_O_3_ was very low; adding it to the slurry reduced the chemical reaction participation of silica sand, thereby decreasing the long-term stability of the slurry. The incorporation of nano-activated alumina greatly promoted the formation of tobermorite 11Å, significantly enhancing the slurry’s high-temperature resistance and macroscopic properties. The results of this study also provided good guidance for the design of high-temperature-resistant cement slurry.

## 4. Conclusions

This study optimized the long-term stability at high temperatures of hydroceramic systems by modifying the Ca/Si ratios and the composition of the slurry. The conclusions drawn are as follows:

(1) The hydroceramic systems had superior long-term stability compared to conventional cement systems. The best long-term stability was found in a slurry with a Ca/Si ratio of 1, composed of silica sand + silica fume + calcium hydroxide + α-alumina + nano-activated alumina. The strength reached 19.1 MPa after 2 days of curing, and 19.6 MPa after 90 days of curing, with no evidence of strength retrogression.

(2) The hydration reaction of α-Al_2_O_3_ was minimal, and its inclusion promoted the formation of a small amount of tobermorite 11 Å, thus leading to a modest improvement in macroscopic properties. However, it also reduced the participation of silica sand in the hydration reaction, generating a negative impact on the long-term stability of the system.

(3) The simultaneous inclusion of α-alumina and nano-activated alumina promoted the formation of a large amount of tobermorite 11 Å, thereby significantly enhancing the macroscopic properties of the hydroceramic systems.

(4) The addition of silica fume promoted the reaction of silica sand, and increased the content of C-(A)-S-H in the hydration products, which enhanced the macroscopic properties and long-term stability of the hydroceramic systems.

(5) The slurries with a Ca/Si ratio of 0.5 exhibited superior macroscopic properties after short-term curing (2 days) compared to the slurries with a ratio of 1. However, there was significant strength retrogression after long-term curing (90 days), primarily due to the formation of increased amounts of reyerite during prolonged curing.

(6) It can be concluded that slurries within hydroceramic systems that maintain stable strength are characterized by the early formation of a large amount of tobermorite 11 Å with stable content and the complete reaction of silica sand in the early stages with no significant new mineral formation under long-term curing. The primary reasons for the decline in thlong-term stability of some slurries mainly included an increase in internal pore size, the partial conversion of C-(A)-S-H into crystalline minerals, and the transformation of some tobermorite into xonotlite.

## Figures and Tables

**Table 1:** The composition, particle size, and density of raw materials.

Composition	CH	SL	SF	α-Al	Nano-Al
Al_2_O_3_		0.324	0.564	99.598	97.281
CaO	98.404	0.029	0.577	0.045	0.024
Fe_2_O_3_	0.077	0.028	0.097	0.018	0.025
K_2_O	0.222	0.027	0.627	0.007	0.004
MgO	0.955			0.125	0.175
Na_2_O					
SO_3_	0.016		0.612	0.034	
P_2_O_5_					
Cl		0.011	0.058	0.019	2.430
SrO	0.041		0.007		
SiO_2_	0.257	99.556	97.426	0.065	
Density (g/cm^3^)	2.260	2.630	2.360	4.050	4.290
D_50_ (μm)	4.190	14.200	1.960	3.380	0.214

**Table 2:** Detailed composition design of the slurries (% by weight of calcium hydroxide).

Slurry	Ca/Si/Al	SL	SF	CH	α-Al	Nano-Al	SUS	DIS	RET	FLU	DEF
L-CS	1:2	162.1	0.0	100.0	0.0	0.0	0.9	1.3	6.6 *	7.9	0.9
L-CSA	1:2:1	162.4	0.0	100.0	69.1	0.0	2.2	1.7	8.3 *	9.9	1.1
L-CSAA	1:2:1	162.4	0.0	100.0	60.8	8.3	2.5	1.7	16.6 *	9.9	1.1
L-CSS	1:2	135.9	26.2	100.0	0.0	0.0	0.9	1.3	10.5 *	7.9	0.9
L-CSSA	1:2:1	129.3	33.1	100.0	69.1	0.0	1.9	1.7	9.9 *	9.9	1.1
L-CSSAA	1:2:1	129.3	33.1	100.0	60.8	8.3	1.9	1.7	19.9 *	9.9	1.1
H-CS	1:1	81.1	0.0	100.0	0.0	0.0	0.2	4.5	7.2 *	5.4	0.6
H-CSA	2:2:1	81.1	0.0	100.0	34.4	0.0	0.6	2.2	8.6 *	6.5	0.7
H-CSAA	2:2:1	81.1	0.0	100.0	29.1	5.4	0.5	2.2	14.0 *	6.5	0.7
H-CSS	1:1	63.0	18.1	100.0	0.0	0.0	0.2	4.5	9.1 *	5.4	0.6
H-CSSA	2:2:1	59.5	21.6	100.0	34.4	0.0	0.4	3.2	10.8 *	6.5	0.7
H-CSSAA	2:2:1	59.5	21.6	100.0	29.1	5.4	0.2	4.3	14.0 *	6.5	0.7

* The dosage of the retarder is an optimized value determined after testing the hydration reaction time of slurries.

**Table 3:** The changes in the amounts of hydration products of slurries L-CS, L-CSA, and L-CSAA at various curing times.

Slurry	Curing Time	AD	SL	α-Al	CH	Nano-Al	FW	T11	T9	XO	OM	AM
L-CS	0 d	1.3	40.3	0.0	24.9	0.0	33.5	0.0	0.0	0.0	0.0	0.0
	2 d	1.3	22.0	0.0	0.0	0.0	29.8	15.4	0.0	13.6	0.0	17.9
	30 d	1.3	21.8	0.0	0.0	0.0	30.1	1.2	7.2	15.9	3.4	19.1
	90 d	1.3	19.9	0.0	0.0	0.0	30.0	0.8	2.5	19.2	0.0	26.3
L-CSA	0 d	1.5	30.6	13.0	18.9	0.0	36.0	0.0	0.0	0.0	0.0	0.0
	2 d	1.5	18.3	11.9	0.0	0.0	31.5	2.8	2.6	3.2	0.8	27.4
	30 d	1.5	15.5	11.7	0.0	0.0	32.0	4.5	3.0	2.1	0.1	29.6
	90 d	1.5	11.2	11.5	0.0	0.0	31.9	1.1	5.5	3.3	5.9	28.1
L-CSAA	0 d	1.9	25.8	12.1	20.0	1.7	38.5	0.0	0.0	0.0	0.0	0.0
	2 d	1.9	14.5	10.5	0.0	0.0	34.8	3.0	2.7	2.0	0.9	29.7
	30 d	1.9	14.3	8.7	0.0	0.0	33.6	4.3	1.6	2.1	1.5	32.0
	90 d	1.9	10.4	8.2	0.0	0.0	33.7	3.0	1.8	2.1	1.2	37.7

Note: AD = additives, SL = silica sand/quartz, α-Al = α-alumina/corundum, CH = calcium hydroxide, Nano-Al = nano-activated alumina, FW = free water, T11 = tobermorite 11 Å, T9 = tobermorite 9 Å, XO = xonotlite, OM = other minerals, AM = amorphous.

**Table 4:** The changes in the amounts of hydration products of slurries L-CSS, L-CSSA, and L-CSSAA at various curing times.

Slurry	Curing Time	AD	SL	α-Al	CH	SF	Nano-Al	FW	T11	T9	XO	RE	OM	AM
L-CSS	0 d	1.5	33.9	0.0	24.9	0.0	6.5	33.2	0.0	0.0	0.0	0.0	0.0	0.0
	2 d	1.5	13.8	0.0	0.0	0.0	0.0	27.8	10.7	0.5	11.4	0.0	3.0	31.3
	30 d	1.5	13.6	0.0	0.0	0.0	0.0	27.9	13.8	0.3	10.0	0.0	1.8	31.1
	90 d	1.5	13.5	0.0	0.0	0.0	0.0	28.3	14.6	0.0	10.3	0.0	3.8	28.0
L-CSSA	0 d	1.5	24.5	13.1	19.0	0.0	6.3	35.6	0.0	0.0	0.0	0.0	0.0	0.0
	2 d	1.5	10.4	12.6	0.0	0.0	0.0	31.3	0.0	3.3	1.4	4.8	1.1	33.6
	30 d	1.5	10.3	10.1	0.0	0.0	0.0	31.4	0.0	1.1	0.9	6.1	0.5	38.1
	90 d	1.5	7.4	9.9	0.0	0.0	0.0	31.5	0.0	0.8	0.2	7.5	0.1	41.1
L-CSSAA	0 d	1.8	24.3	11.4	18.8	1.6	6.2	35.9	0.0	0.0	0.0	0.0	0.0	0.0
	2 d	1.8	13.3	10.8	0.0	0.0	0.0	30.7	4.0	4.0	10.2	0.0	0.5	24.7
	30 d	1.8	13.1	10.6	0.0	0.0	0.0	31.0	3.6	3.4	11.5	0.0	2.1	22.9
	90 d	1.8	9.1	8.7	0.0	0.0	0.0	30.9	0.0	0.0	2.4	8.6	5.1	33.4

Note: AD = additives, SL = silica sand/quartz, α-Al = α-alumina/corundum, CH = calcium hydroxide, SF = silica fume, Nano-Al = nano-activated alumina, FW = free water, T11 = tobermorite 11 Å, T9 = tobermorite 9 Å, XO = xonotlite, RE = reyerite, OM = other minerals, AM=amorphous.

**Table 5:** The changes in the amounts of hydration products of slurries H-CS, H-CSA, and H-CSAA at various curing times.

Slurry	Curing Time	AD	SL	α-Al	CH	Nano-Al	FW	T11	T9	XO	SC	OM	AM
H-CS	0 d	2.6	28.4	0.0	35.0	0.0	34.0	0.0	0.0	0.0	0.0	0.0	0.0
	2 d	2.6	0.1	0.0	0.0	0.0	29.5	0.0	3.0	30.8	6.6	0.0	27.4
	30 d	2.6	0.0	0.0	0.0	0.0	30.2	0.0	3.0	31.5	6.5	0.0	26.2
	90 d	2.6	0.0	0.0	0.0	0.0	29.7	0.2	2.6	32.5	6.5	0.0	25.9
H-CSA	0 d	1.8	24.1	10.2	29.7	0.0	34.2	0.0	0.0	0.0	0.0	0.0	0.0
	2 d	1.8	0.0	10.2	0.0	0.0	29.2	5.2	5.7	18.4	0.0	0.0	29.5
	30 d	1.8	0.0	10.1	0.0	0.0	29.5	5.0	5.4	17.8	0.0	0.0	30.4
	90 d	1.8	0.0	10.1	0.0	0.0	29.3	0.0	6.8	21.1	2.8	2.1	26.0
H-CSAA	0 d	2.0	29.5	8.6	23.9	1.6	34.4	0.0	0.0	0.0	0.0	0.0	0.0
	2 d	2.0	1.6	7.6	0.0	0.0	26.6	19.0	4.4	5.4	0.0	0.0	33.4
	30 d	2.0	0.2	6.8	0.0	0.0	26.9	18.6	5.8	5.5	0.0	0.0	34.3
	90 d	2.0	0.0	6.5	0.0	0.0	27.2	16.4	3.3	7.9	0.0	0.0	36.7

Note: AD = additives, SL = silica sand/quartz, α-Al = α-alumina/corundum, CH = calcium hydroxide, Nano-Al = nano-activated alumina, FW = free water, T11 = tobermorite 11 Å, T9 = tobermorite 9 Å, XO = xonotlite, SC = scawtite, OM = other minerals, AM = amorphous.

**Table 6:** The changes in the amounts of hydration products of slurries H-CSS, H-CSSA, and H-CSSAA at various curing times.

Slurry	Curing Time	AD	SL	α-Al	CH	SF	Nano-Al	FW	T11	T9	XO	SC	OM	AM
H-CSS	0 d	2.7	22.1	0.0	35.0	0.0	6.3	33.9	0.0	0.0	0.0	0.0	0.0	0.0
	2 d	2.7	0.1	0.0	0.0	0.0	0.0	29.2	0.7	1.1	33.4	4.7	0.3	27.8
	30 d	2.7	0.1	0.0	0.0	0.0	0.0	29.5	0.7	0.0	31.9	4.5	0.3	30.3
	90 d	2.7	0.1	0.0	0.0	0.0	0.0	29.7	0.7	0.0	31.7	4.5	0.4	30.2
H-CSSA	0 d	2.1	17.7	10.3	29.8	0.0	6.4	33.7	0.0	0.0	0.0	0.0	0.0	0.0
	2 d	2.1	0.0	10.0	0.0	0.0	0.0	28.0	1.7	3.5	22.9	0.0	0.3	31.5
	30 d	2.1	0.0	9.9	0.0	0.0	0.0	28.4	1.3	4.9	22.1	0.0	1.2	30.1
	90 d	2.1	0.0	8.0	0.0	0.0	0.0	28.5	2.4	3.2	16.6	0.0	0.8	38.4
H-CSSAA	0 d	2.6	17.6	8.6	29.6	1.6	6.4	33.6	0.0	0.0	0.0	0.0	0.0	0.0
	2 d	2.6	0.0	8.4	0.0	0.0	0.0	25.9	12.8	0.0	5.6	0.0	0.0	44.7
	30 d	2.6	0.0	6.7	0.0	0.0	0.0	26.2	15.0	0.0	4.6	0.0	2.4	42.5
	90 d	2.6	0.0	6.7	0.0	0.0	0.0	25.8	14.7	0.0	3.7	0.0	2.8	43.7

Note: AD = additives, SL = silica sand/quartz, α-Al = α-alumina/corundum, CH = calcium hydroxide, SF = silica fume, Nano-Al = nano-activated alumina, FW = free water, T11 = tobermorite 11 Å, T9 = tobermorite 9 Å, XO = xonotlite, SC = scawtite, OM = other minerals, AM = amorphous.

## Data Availability

The original contributions presented in the study are included in the article, further inquiries can be directed to the corresponding author.
